# Photochemical Degradation of the UV Filter Octyl Methoxy Cinnamate Probed via Laser-Interfaced Mass Spectrometry

**DOI:** 10.3390/molecules27248796

**Published:** 2022-12-12

**Authors:** Natalie G. K. Wong, Maria Sereli, Cate S. Anstöter, Caroline E. H. Dessent

**Affiliations:** Department of Chemistry, University of York, Heslington YO10 5DD, UK

**Keywords:** sunscreens, photodynamics, cinnamates, mass spectrometry, lasers, photodegradation

## Abstract

Octyl methoxycinnamate (OMC) is a common UVA and UVB filter molecule that is widely used in commercial sunscreens. Here, we used gas-phase laser photodissociation spectroscopy to characterise the intrinsic photostability and photodegradation products of OMC by studying the system in its protonated form, i.e., [OMC·H]^+^. The major photofragments observed were *m*/*z* 179, 161, and 133, corresponding to fragmentation on either side of the ether oxygen of the ester group (*m*/*z* 179 and 161) or the C–C bond adjacent to the ester carbonyl group. Additional measurements were obtained using higher-energy collisional dissociation mass spectrometry (HCD-MS) to identify fragments that resulted from the breakdown of the vibrationally hot electronic ground state. We found that the *m*/*z* 179 and 161 ions were the main fragments produced by this route. Notably, the *m*/*z* 133 ion was not observed through HCD-MS, revealing that this product ion is only produced through a photochemical route. Our results demonstrate that UV photoexcitation of OMC is able to access a dissociative excited-state surface that uniquely leads to the rupture of the C–C bond adjacent to the key ester carbonyl group.

## 1. Introduction

Commercial sunscreens are an important public health product since they provide crucial protection from damaging ultraviolet solar radiation and hence skin cancer [1,2,3]. The effectiveness of an organic sunscreen chromophore, one of the key components of sunscreen mixtures, is determined both by its intrinsic ability to absorb UV photons and the extent to which it is photostable following exposure to light [4,5]. A sunscreen chromophore that is unstable over a short-to-medium timescale will clearly be of limited use in sunscreen formulations. Additionally, it is undesirable for sunscreen to degrade into either reactive or toxic products [6]. A final point of consideration is the emergence of sunscreen agents as a pollutant of natural water environments, for which knowledge of degradation products is critical to understanding the impact on ecosystems [7,8]. These combined considerations mean that it is important to (i) determine the factors that affect the photostability of individual sunscreen chromophores and (ii) gain a broader fundamental understanding of the molecular level factors that affect photostability [9].

**Figure 1:**
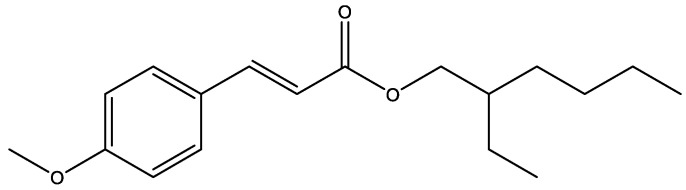
Chemical structure of *trans*-OMC.

Octyl methoxycinnamate (OMC: alternatively known as ethylhexyl methoyxycinnamate and octinoxate) is a common UV filter used in a variety of sunscreen products [10]. The chemical structure of OMC is illustrated in Figure 1. It is generally employed as a UVB (280–320 nm) filter, although the low-energy tail of its absorption spectrum extends into the UVA (320–400 nm). OMC exists in the forms of *trans* and *cis* isomers, both of which are important in their photochemical behaviours. For the *trans* isomer, the main photochemical reaction pathway in solution is isomerization to the *cis* form. The *cis* form has a lower extinction coefficient than the *trans* form, so the overall absorptivity of OMC decreases under UV irradiation [11]. A range of experimental techniques (e.g., steady state, time-resolved, Raman, etc.) have been used to study OMC’s excited-state photodynamics [6,12,13,14,15,16,17] and reactive oxygen sensitization, with quantum chemical studies also exploring details of the excited-state characteristics [18,19,20]. Intriguingly, a wide difference in the reduction of OMC absorptivity has been reported in the various experimental studies, with absorbance being observed to vary greatly under different experimental conditions. While some studies have reported that OMC degrades irreversibly in solution, losing up to 90% of its absorbance [21], others have found that it forms a photostationary state between the *trans* and *cis* isomers with only 30–50% loss of absorption [11,22,23].

A recent extensive study by Bardeen and co-workers on UV excitation of OMC found that aggregation was important in its photochemical behaviour, with OMC aggregation leading to a complex mixture of photoproducts (truxillic and truxinic dimers), including the production of UVA photoproducts, which generated singlet oxygen [22]. Given that aggregation effects are implicated in that work, it is useful to consider using gas-phase measurements to investigate the intrinsic photochemical properties of OMC as a basis for future studies of the aggregates. Our group has employed laser-interfaced mass spectrometry (LIMS) to perform such gas-phase investigations on a range of organic sunscreen molecules and complexes, demonstrating the utility of this approach for characterising photostability and mapping photodegradation pathways [24,25,26,27].

In this study, we applied LIMS to probe OMC’s intrinsic photochemistry and photodegradation pathways by studying OMC in its protonated form, i.e., [OMC·H]^+^, to allow for its study via LIMS. In addition to the gas-phase laser photodissociation data, higher-energy collision-induced (HCD) fragmentation data are also presented. These complementary data aid in the interpretation of the OMC mass spectra and provides insight into its photodynamics. Importantly, the complementary collisional and photoexcitation dissociation measurements allow us to identify photochemical products that are generated by long-lived, dissociative excited states.

## 2. Results

### 2.1. Gas-Phase Absorption Spectrum of Protonated OMC

**Figure 2:**
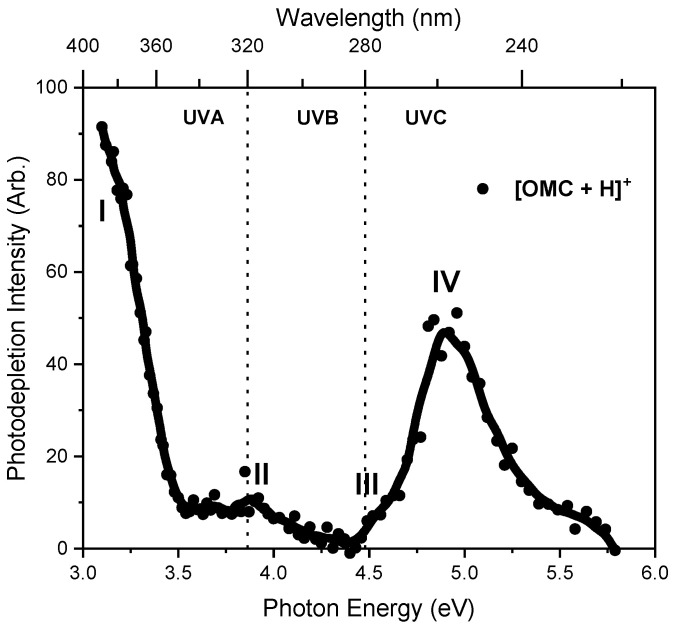
Gas-phase UV absorption (photodepletion) spectrum of [OMC·H]^+^. The solid line is a five-point adjacent average of the data points.

The photodepletion (gas-phase absorption) spectrum of mass-selected [OMC·H]^+^ (*m/z* 291) across the 3.10–5.79 eV (400–214 nm) range is shown in Figure 2. Mass selection is a key advantage of laser-interfaced photodissociation mass spectrometry since it allows us to directly probe the intrinsic properties of isolated monomeric charged systems. The gas-phase absorption spectrum displays very strong absorption in the UV-A region (with strong absorption below our lowest accessible measured photon energy of 3.1 eV) through band I which then reduces in intensity through the higher UV-B region (3.6–4.5 eV) of band II. Absorption again increases above 4.5 eV through band III and into the higher UVC region (IV). OMC is primarily used as a UVB absorber in sunscreen formulations [10] with λ_max_ = 310 nm, so it is notable that the strong band I absorption feature appears significantly red-shifted for the protonated gas-phase species. A similar red shift was observed for protonated oxybenzone [27], where protonation was found to occur in the aromatic conjugated carbonyl group, as is also expected for OMC⋅H^+^.

### 2.2. Gas-Phase Absorption Spectrum of Photofragment Ions of [OMC·H]^+^

**Figure 3:**
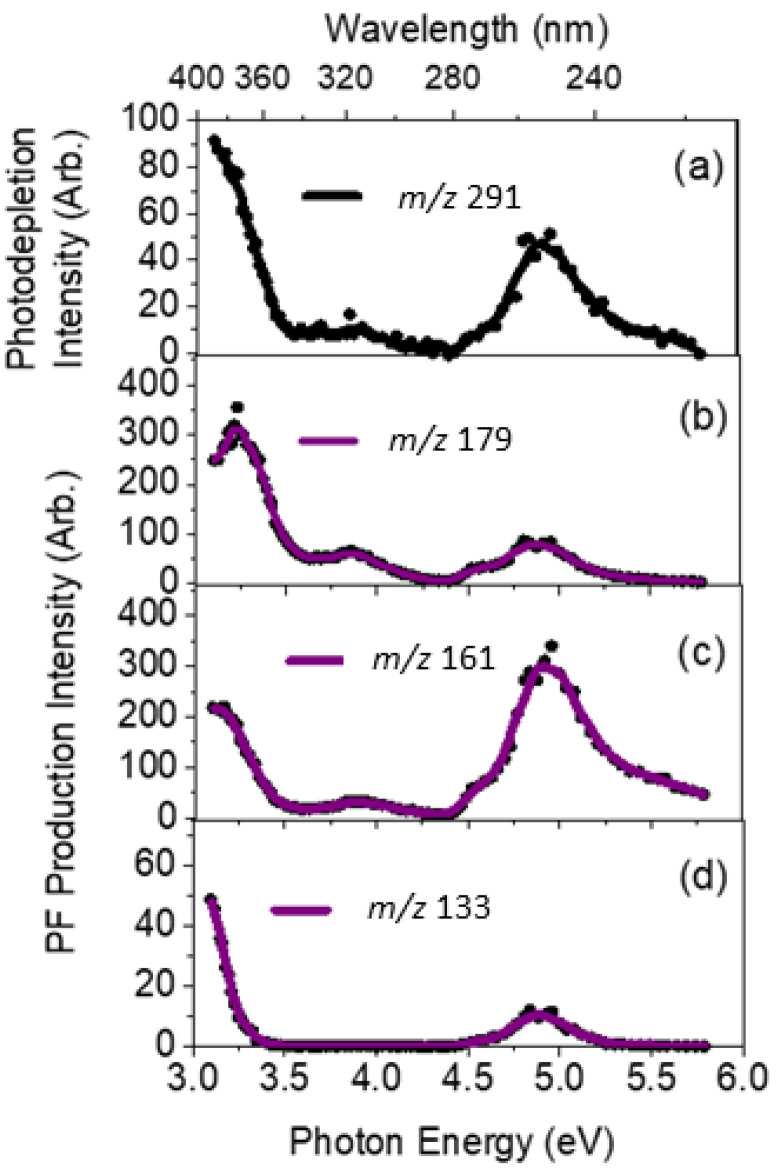
(**a**) Gas-phase UV absorption (photodepletion) spectrum of [OMC+H]^+^. The complementary photofragment production intensity spectra are shown for the three major photofragments with (**b**) *m/z* 179, (**c**) *m*/*z* 161, and (**d**) *m*/*z* 133, respectively. The solid line is a five-point adjacent average of the data points.

We next turn to the photofragment ions produced following photoabsorption by OMC⋅H^+^. These photofragment production spectra were produced concurrently with the gas-phase absorption spectrum (Figure 2) across the full laser excitation region scanned. Figure 3 displays the production spectra of the three most intense photofragments of [OMC·H]^+^, which correspond to *m*/*z* 179, 161, and 133 (Figure 3b–d). The photodepletion spectrum of [OMC·H]^+^ (*m*/*z* 291) is also again displayed for ease of comparison in Figure 3a. While the intensity of the photofragments varied with photon energy, their production profiles are similar to the parent ion photodepletion, with each of these fragments being produced strongly in the UVA and UVC regions but with relatively low intensity in the UVB region. This general production profile mirrors the photodepletion spectrum (Figure 3a) and is consistent with these ions being produced through photoexcitation processes.

The *m*/*z* 179 fragment can be assigned as protonated 4-methoxy cinnamic acid, with the *m*/*z* 161 fragment corresponding to cationic 4-methoxycinnamaldehyde, where the positive charge is likely located on the aldehyde oxygen atom following the loss of a C_8_H_17_O neutral fragment from OMC⋅H^+^. These products are in line with photoproducts of OMC observed previously by MacManus Spencer et al. in a study where solutions of OMC were photolyzed with UV light and photoproducts identified by HPLC [28]. This gives us confidence that the gas-phase experiments on protonated OMC are directly relevant to the photolysis of OMC in the condensed phase. The *m*/*z* 161 and 179 pair of fragments correspond to differential fission of the C–O ether bond of the ester group of OMC. Proposed structures for the three major photofragments are presented in Table 1. A number of less intense photofragments were also observed: The production spectra for these photofragments can be found in Section S2 of the Supplementary Materials.

**Figure 4:**
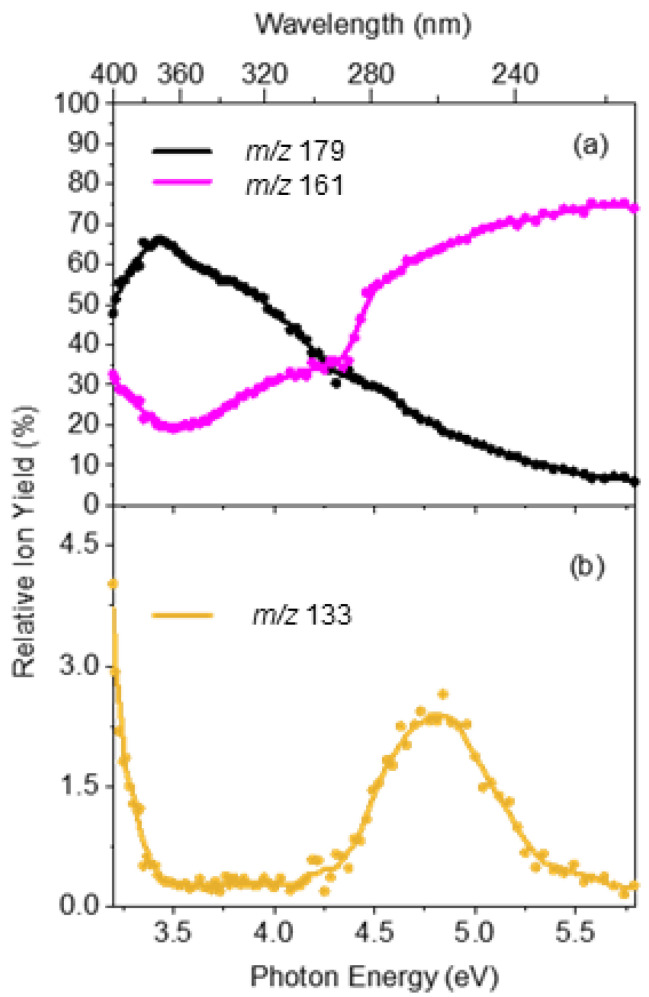
Relative ion yield plot for the (**a**) major photofragments *m*/*z* 179 and *m*/*z* 161 and the (**b**) minor photofragment *m*/*z* 133 of [OMC·H]^+^ for photon energies between 3.2 and 5.8 eV. The solid line is a five-point adjacent average of the data points.

Figure 4 displays the relative ion yields of the major photofragments as a function of photon energy to provide a more concise overview of photofragment production as a function of excitation energy. Overall, the relative ion yields of the major photofragments at *m*/*z* 179 and 161 are considerably greater than that of the minor *m*/*z* 133 photofragment, as is evident in the ion intensity values displayed in Figure 4. The ion yield for the *m*/*z* 179 photofragment peaks in the UVA region (3.2–3.6 eV) and then decreases smoothly across the UVB and UVC regions whereas the yield for *m*/*z* 169 shows the opposite behaviour. The lower *m*/*z* photofragment, *m*/*z* 161, increases over the higher energy region while the higher *m*/*z* photofragment, *m*/*z* 179, decreases concomitantly. This suggests that the *m*/*z* 161 fragment forms via a secondary fragmentation route from the primary *m*/*z* 179 fragment through the loss of a neutral water molecule as the internal energy of *m*/*z* 179 increases. Importantly, it is evident from the ion yield plot that the *m*/*z* 133 photofragment has a unique production profile, indicating that this photofragment is produced through a distinct photochemical pathway(s). We note that this fragment corresponds to the fission of the C2–C3 bond between the alkene and carbonyl groups of the side chain. Finally, we note that we see other photochemical fragments that are produced at a lower intensity than *m*/*z* 133 (Figures S2 and S3). Interestingly, the *m*/*z* 121 fragment displays a distinctive production profile across the 3.2–4.2 eV range, suggesting a distinct production route from other photofragments (Figure S3).

### 2.3. Comparison of Photofragmentation and HCD Fragmentation of [OMC·H]^+^

An efficient sunscreen filter needs to be able to return to the ground state from the excited state by releasing the excess energy gained through UV photon absorption as heat into the surroundings [1]. In the solution phase, this can happen through vibrational relaxation following the transfer of the excited-state decay. In the gas phase, no solvent is present and therefore excess energy is retained by the molecule, so it fragments following relaxation back to the vibrationally hot electronic ground state. To probe the identity of the fragments produced from a vibrationally hot ground state of [OMC·H]^+^, HCD experiments were conducted. HCD allows the characterization of the ground-state fragmentation pathways as a function of increasing internal energy, thus providing information on the electronic ground state of a system as a function of internal energy [7]. Furthermore, it also allows for the identification of secondary fragments, which are produced from primary fragments with a high internal energy.

**Figure 5:**
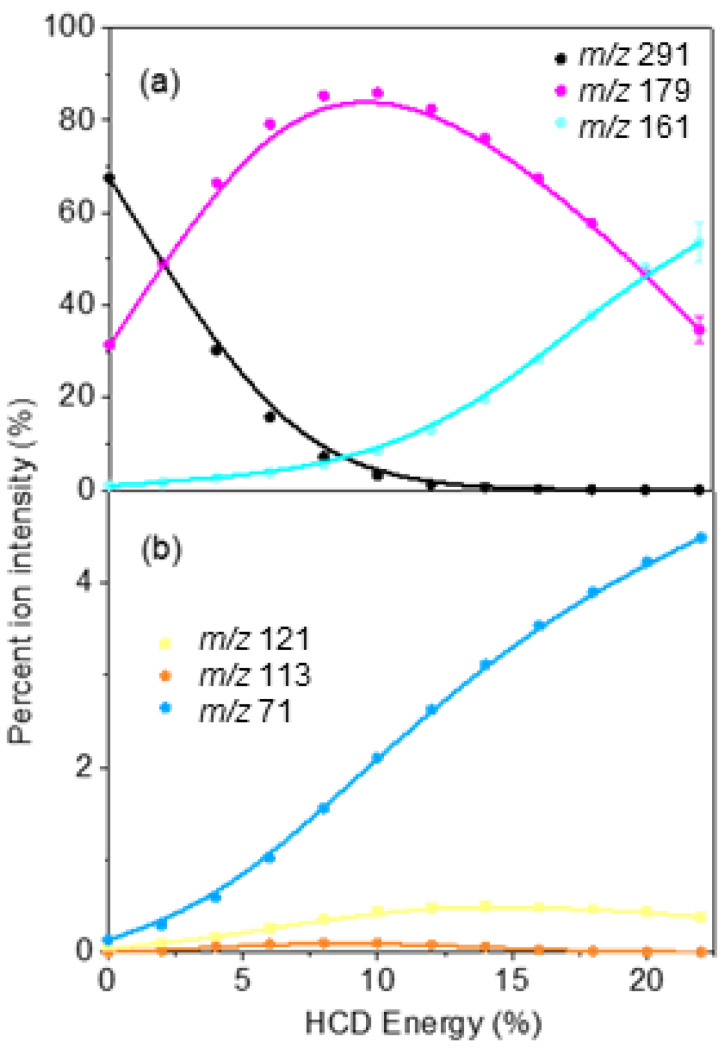
Percent ion intensity of the (**a**) major and (**b**) minor thermal fragments of [OMC·H]^+^ as a function of HCD energy. The solid line is a three-point adjacent average of the data points.

Figure 5 displays the HCD results for [OMC·H]^+^, with Figure 5a displaying the highest intensity HCD fragments, *m*/*z* 179 and *m*/*z* 161, while Figure 5b displays the series of minor HCD fragments, *m*/*z* 121, 113, and 71. The production profile of the *m*/*z* 179 and *m*/*z* 161 fragments indicate that *m*/*z* 179 is produced at the lowest collisional energies but fragments with increasing cross-section into *m*/*z* 161 as internal energy increases through the loss of a neutral water molecule. This allows us to assign *m*/*z* 161 as a secondary fragment of *m*/*z* 179. It is notable that the production profile of the minor fragment *m*/*z* 71 also increases strongly with increasing collision energy, indicating that it is also a secondary fragment ion. We note that the *m*/*z* 133 photofragment (Figure 3d) is not observed as a collisional fragment via HCD, indicating that there is no pathway to its production through the breakdown of the vibrationally hot ground electronic state of [OMC⋅H^+^].

## 3. Discussion

As for other systems we have studied with the complementary LIMS and HCD methods, our results provide insight into the intrinsic photodynamics of the system studied. To provide some brief background, for molecular systems where the photofragments produced correlate with fragments produced from a hot ground state (i.e., the HCD fragments), the photodecay of the excited state is described as a “statistical decay” process [29]. In contrast, if dissociation occurs directly from the excited state without the involvement of a conical intersection to return the system to a near-starting point geometry, a “non-statistical” decay process is occurring [30]. In non-statistical decay, the photofragments obtained will be significantly different in terms of their identities and relative intensities from the hot ground electronic-state fragments. Therefore, fragments that are produced only through UV excitation are termed non-statistical (non-ergodic) and can be identified as being produced directly from the excited state surface [31].

As noted above, the *m*/*z* 133 photofragment (Figure 3b) was not observed as a thermal fragment via HCD. The ion yield production spectrum of *m*/*z* 133 (Figure 4b) shows clearly that its production peaked around 4.8 eV with a profile that is distinctive to that of the other major photofragments. Taken together, these observations support the assignment of *m*/*z* 133 as a photochemical product that is produced directly through a dissociative, longer lived e.g., triplet excited, state. The two most intense photofragments (*m*/*z* 179 and *m*/*z* 161) were the same as the main HCD fragments, in line with photodissociation of [OMC·H]^+^ being primarily statistical. However, the presence of the minor dissociative decay channel provided a destructive loss channel for OMC that led to enhanced UV degradation over time.

These conclusions are in broad agreement with previous research on the photodynamics of OMC. Tan and coworkers studied OMC’s photodynamics in the gas phase by combining two-colour resonance-enhanced two-photon ionisation (RE2PI) and UV–UV depletion spectroscopy [10]. They found that UV ^1^ππ* excitation was followed by relaxation to an “optically dark” ^1^nπ* state before transfer to a triplet state and then the ground state, (S_0_) so that the process of overall excited state decay was slower than for an ideal sunscreen. Ebata et al. [32] subsequently used laser-induced fluorescence (LIF) spectroscopy, UV−UV hole-burning (HB) spectroscopy as well as density functional theory (DFT) calculations to further investigate the non-radiative decay followed by OMC. They also defined the photodecay process of OMC as involving multistep internal conversions and intersystem crossings, i.e., (S_1_ (*trans*, ^1^ππ*) → ^1^nπ* → T_1_ (^3^ππ*) → S_0_ (*cis*). The triplet state was found to have a lifetime of 20 ns in this study. The novelty of our experimental method is the identification of the photoproduct that was produced through the extended excited-state lifetime. Such dissociative pathways are critically important in determining sunscreen photostability since they destroy UV absorption and potentially create harmful photoproducts, such as free radicals.

## 4. Materials and Methods

**General.** OMC (98% purity) was purchased from Sigma Aldrich (St. Louis, MO, USA) and was used without further purification. HPLC-grade ethanol was purchased from Fisher Scientific, Inc. (Pittsburgh, PA, USA) and again used as received.

**Laser-Interfaced Mass Spectrometry.** The experiment has been described in-full previously; for a full description including a schematic, we direct readers to reference [33]. Briefly, gas-phase UV photodissociation experiments were conducted in an AmaZon SL dual funnel electrospray ionization quadrupole ion trap (ESI-QIT) mass spectrometer (Bruker Daltonics Inc., Billerica, MA, USA), which was modified to allow for laser-interfaced mass spectrometry, as described previously [34]. Solutions of OMC (1.0 × 10^−4^ mol dm^−3^ with trifluoroacetic acid to aid ionization) in EtOH were introduced to the mass spectrometer via ESI using typical instrumental parameters: nebulizing gas pressure of 10.0 psi, an injection rate of 0.33 mL/h, a drying gas flow rate of 8.0 L/min, and run in the negative ion mode at a capillary temperature of 160 °C to form their deprotonated species. Small amounts of trifluoroacetic acid were added to the OMC solution to enhance ionization efficiency.

OMC⋅H^+^ ions were mass selected and isolated in the ion trap prior to UV laser irradiation. UV–Vis photons were produced with a 10 Hz Nd:YAG (Surelite™, Amplitude Laser Group, San Jose, CA, USA) -pumped OPO laser (Horizon™, Amplitude Laser Group, San Jose, CA, USA), providing ~0.3 mJ across the range 400–214 nm (3.10–5.79 eV). Laser step sizes of 2 nm were used throughout. The laser beam was focused as described previously [34]. Photofragmentation experiments were conducted with a set ion accumulation time of 10 ms and a corresponding fragmentation time of 100 ms, allowing for each mass-selected ion packet to interact with one laser pulse and minimize the likelihood of multiphoton events. When fluorescence is negligible, UV-excited gaseous ions fragment upon excited-state relaxation, yielding an action absorption spectrum by photodepletion [34,35,36].

**Analysis.** In line with previous analysis by our group, as detailed in previous publications [33], the photodepletion (PD) of [OMC+H]^+^ was measured as a function of scanned wavelength, with the photofragment production (PF) recorded simultaneously at each corresponding wavelength.

(1) $$\mathrm{Photodepletion}\;\mathrm{Intensity}=\frac{\ln(\frac{{\mathrm{Int}}_{\mathrm{OFF}}}{{\mathrm{Int}}_{\mathrm{ON}}})}{\lambda \;\times \;P}$$

(2) $$\mathrm{Photofragment}\;\mathrm{Production}\;\mathrm{Intensity}=\frac{\ln(\frac{{\mathrm{Int}}_{\mathrm{FRAG}}}{{\mathrm{Int}}_{\mathrm{OFF}}})}{\lambda \;\times \;P}$$

As described by Equations (1) and (2), Int_OFF_ and Int_ON_ represent the parent ion intensities with laser off and on, respectively; Int_FRAG_ is the photofragment intensity with the laser on; λ is the excitation wavelength (nm); and P is the tuneable laser pulse energy (mJ). The photodepletion intensities were taken from an average of three repeat runs at each wavelength within the range studied. We note that photofragment ions with *m*/*z* < 50 are not detectable within our mass spectrometer since low masses fall outside of the mass window of the ion trap.

**Collision-Induced Dissociation.** Higher-energy collisional dissociation (HCD) of OMC⋅H^+^ was performed on the Thermo Fisher Orbitrap Fusion^TM^ mass spectrometer with the following settings: the syringe was operated at a flow rate of 3 μL/min and with the following settings: MS2 scan isolation mode, ion trap; detector type, ion trap; positive ion spray voltage (3500 V); negative ion spray voltage (2800 V); RF lens (60%); normalized AGC target (100%); maximum injection time (100 ms); ion transfer tube temperature (275 °C); and vaporizer temperature (20 °C). For the MS scan in this instrument, the settings were as follows: detector type, Orbitrap; positive ion spray voltage (3200 V); negative ion spray voltage (2500 V); RF lens (45%); normalized AGC target (100%); and maximum injection time (100 ms).

## 5. Conclusions

Laser-interfaced mass spectrometry of [OMC·H]^+^ was used to obtain its gas-phase absorption spectrum via photodepletion spectroscopy along with the accompanying photofragment production spectra. Upon photoexcitation, [OMC·H]^+^ was observed to fragment primarily into the *m*/*z* 179 and *m*/*z* 161 fragments, in line with the main known photofragments of neutral OMC (4-methoxy cinnamic acid and 4-methoxycinnamaldehyde). HCD measurements were performed to identify the vibrationally hot electronic ground state fragments of [OMC·H^+^]. The *m*/*z* 179 and *m*/*z* 161 ions were found to be the major thermal fragments, matching the major photofragments observed, which revealed that the predominant photofragmentation pathways are statistical for the [OMC·H^+^] ion.

The minor photofragment, *m*/*z* 133, was not observed as an HCD fragment, indicating that the fragmentation pathway was accessed purely through photochemical excitation. The distinctive nature of this photofragment is also evident in its unique production profile, which is shown very clearly in the ion yield spectrum displayed in Figure 4b. Production of fragments with this signature profile has previously been associated with the excitation of long-lived triplet excited states, e.g., as in deprotonated 2-phenylbenzimidazole-5-sulfonic acid where such photochemical photoproducts were associated with excitation of the T_1_ state responsible for photosensitization [31]. Our observations indicate that such a state may be accessed between excitation energies of 410–240 nm for OMC and into the strong UVA region. This is in line with the known property of OMC to generate singlet oxygen under UVA irradiation and previous characterisation of the photodynamics of OMC that revealed the involvement of a T_1_ state along the excitation decay pathway. Our results are novel, as the actual photoproduct (C2–C3 side-chain fission) has been identified here in a straightforward manner via the mass spectrometry functionality of our experimental method. This is important, as it can allow the potential toxicity of a final degradation product to be assessed but also because it can provide insight into the detailed mechanisms for the formation of the total suite of decay products [27]. While a detailed analysis of the photodegradation pathways and photoproducts is beyond the scope of this paper, we note that a number of previous studies have investigated the breakdown of OMC in other mediums [15,16]. Although the degradation processes may vary as a result of their chemical environment, it is often found that there are similar intermediates and degradation products. The results presented in this study further illustrate the utility of LIMS photodissociation measurements as a straightforward route for identifying photoproducts [37].

## Figures and Tables

**Table 1:** Proposed structures for the fragments obtained via higher-energy collisional dissociation (HCD) and laser photoexcitation of [OMC·H]^+^.

Fragment Mass (*m*/*z*)	Proposed Fragment Structure	∆*m*/*z* ^1^	Observed HCD Fragment (Y/N) ^2^	Observed Photofragment (Y/N) ^2^
179	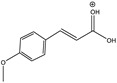	112	Y (s)	Y (vs)
161	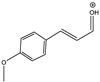	130	Y (s)	Y (vs)
133	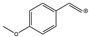	158	N	Y (vw)
121	-	170	Y (w)	Y (vw)
113	-	178	Y (vw)	N
71	-	220	Y (vw)	Y (vw)

^1^ difference in energy between the parent fragment [OMC·H]^+^ (*m*/*z* 291) and the mass of the neutral fragment lost. ^2^ very strong (vs), strong (s), weak (w), and very weak (vw).

## Data Availability

The data presented in this study are available from the authors on request. OMC is available to purchase from Sigma Aldrich.

## Supplementary Materials

The following supporting information can be downloaded at https://www.mdpi.com/article/10.3390/molecules27248796/s1, Section S1: Raw ion intensity mass spectra of the thermal fragments of [OMC+H]^+^, Section S2: Photofragment production intensity of the minor photofragments produced by [OMC+H]^+^ obtained via laser-interfaced mass spectrometry.
