# Supplementary material for: Photochemical Degradation of the UV Filter Octyl Methoxy Cinnamate Probed via Laser-Interfaced Mass Spectrometry
Source: Molecules. 2022 Dec 12;27(24):8796. doi: 10.3390/molecules27248796 (PMC9780824; doi:10.3390/molecules27248796)

molecules

Supplementary Material

Photochemical Degradation of the Sunscreen Octyl methoxycinnamate Probed via Laser Interfaced Mass Spectrometry

Natalie G. K. Wong, Maria Sereli, Cate S. Anstöter and Caroline E. H. Dessent*

Department of Chemistry, University of York, Heslington, YO10 5DD, U. K.
* E-mail: caroline.dessent@york.ac.uk

Content

S1: Raw ion intensity mass spectra of the thermal fragments of $[OMC+H]^+$
S2: Photofragment production spectra of the minor photofragments produced by $[OMC+H]^+$ obtained via laser-interfaced mass spectrometry

S1: Raw ion intensity mass spectra of the thermal fragments of $[OMC+H]^+$

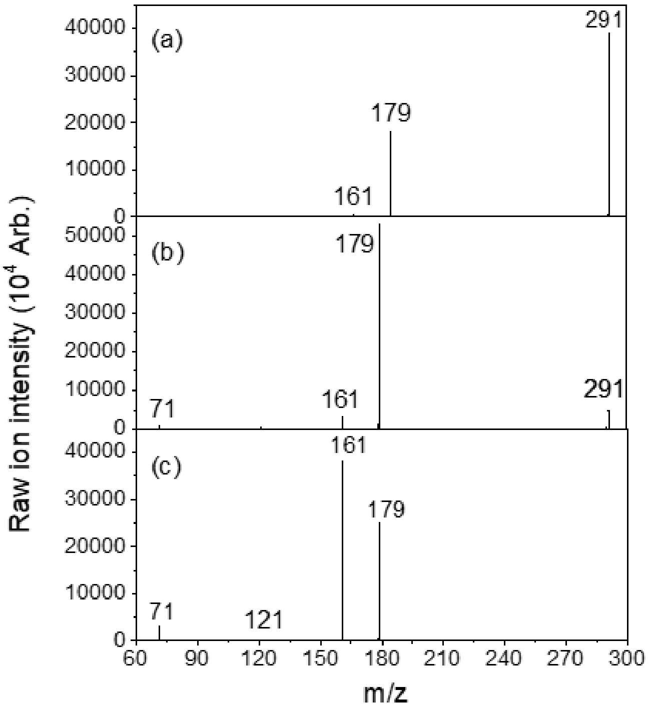

Figure S1: Raw ion intensity mass spectra of the thermal fragments of $[OMC+H]^+$ at 0 % (a), 8 % (b) and 22 % (c) HCD energy.

S2: Photofragment production spectra of the minor photofragments produced by $[OMC+H]^+$ obtained via laser-interfaced mass spectrometry

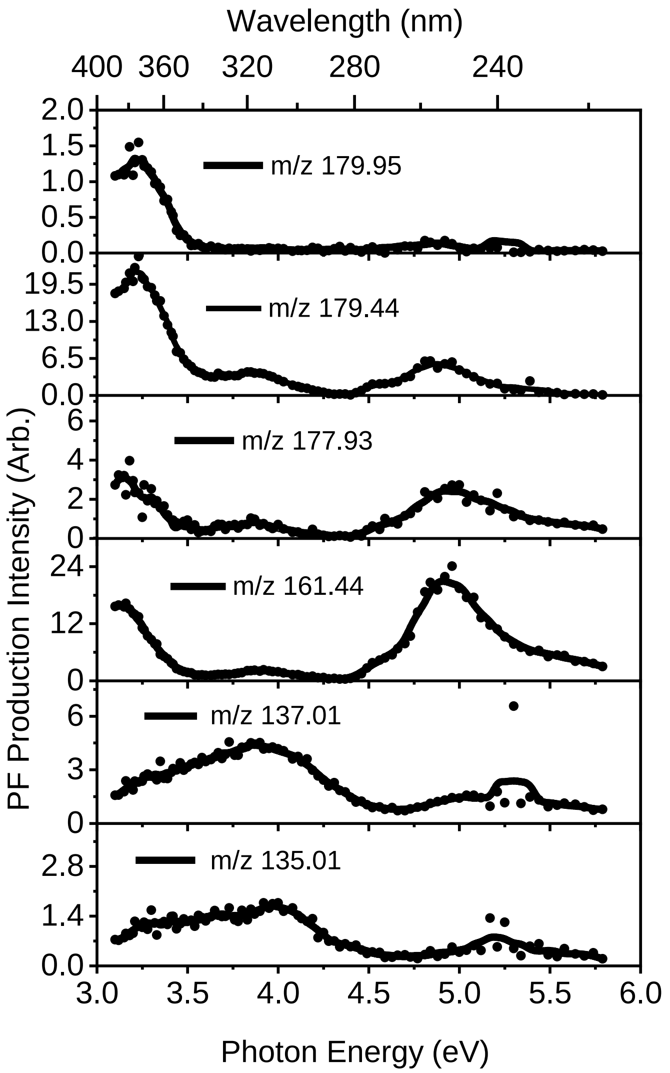

Figure S2: Photofragment production spectra of the minor fragments (*m/z* 179.95, 179.44, 177.93, 161.44, 137.01 and 135.01) of gas-phase $[OMC+H]^+$. The solid line is a five-point adjacent average of the data points.

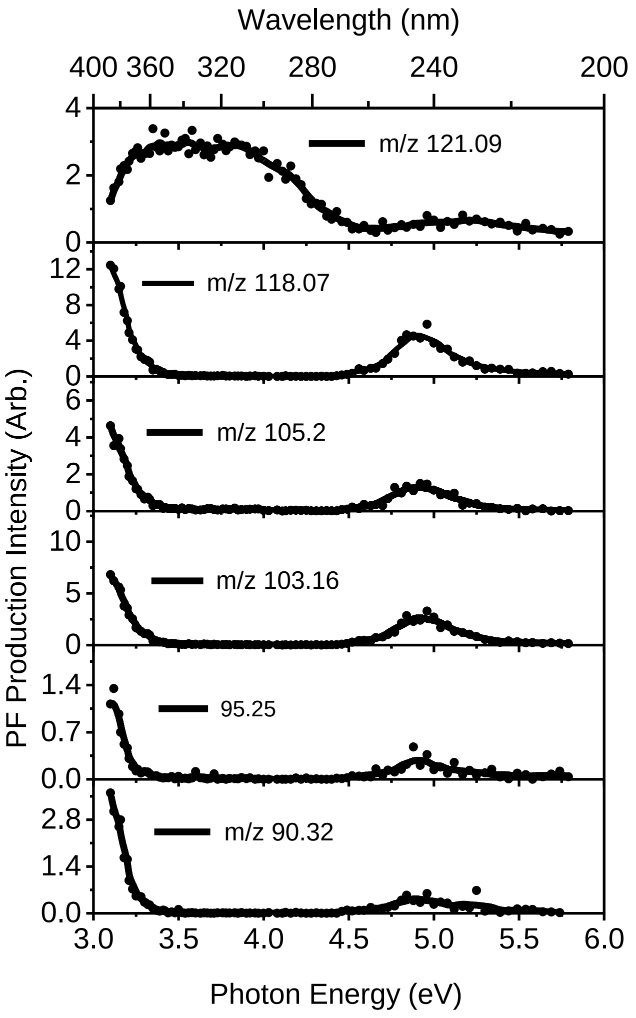

Figure S3: Photofragment production spectra of the minor fragments (*m/z* 121.09, 118.07, 105.2, 103.16, 95.25 and 90.32) of gas-phase [[OMC+H]$^+$. The solid line is a five-point adjacent average of the data points.

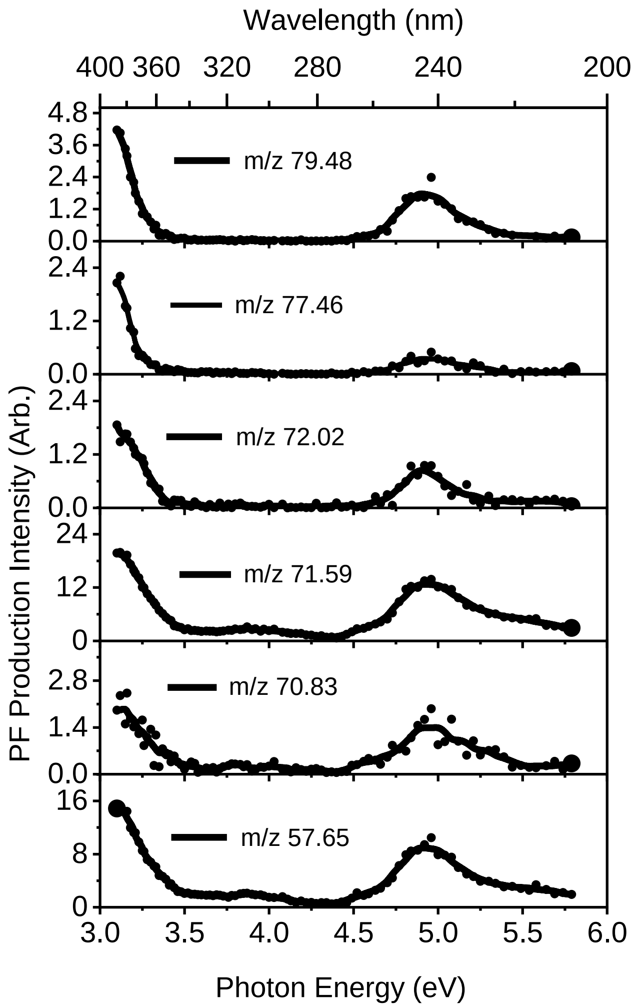

Figure S4: Photofragment production spectra of the minor fragments (*m/z* 79.48, 77.46, 72.02, 71.59, 70.83 and 57.65) of gas-phase $[OMC+H]^+$. The solid line is a five-point adjacent average of the data points.

TOC:

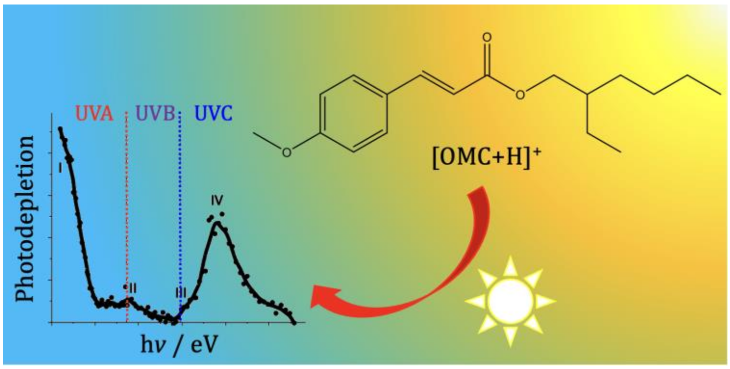

Supplement: Supplementary file 1 [file molecules-27-08796-s001.zip › molecules-2082207-supplementary.pdf]
